# Tribute to health workers in China: A group of respectable population during the outbreak of the COVID-19

**DOI:** 10.7150/ijbs.45135

**Published:** 2020-03-15

**Authors:** Yu-Tao Xiang, Yu Jin, Yu Wang, Qinge Zhang, Ling Zhang, Teris Cheung

**Affiliations:** 1 Unit of Psychiatry, Institute of Translational Medicine, Faculty of Health Sciences, University of Macau, Macao SAR, China;; 2 Center for Cognition and Brain Sciences, University of Macau, Macao SAR, China;; 3 Department of Nursing, Chinese Academy of Medical Sciences - Peking Union Medical College, Peking Union Medical College Hospital, Beijing, China;; 4 The National Clinical Research Center for Mental Disorders & Beijing Key Laboratory of Mental Disorders, Beijing Anding Hospital & the Advanced Innovation Center for Human Brain Protection, Capital Medical University, Beijing, China;; 5 School of Nursing, Hong Kong Polytechnic University, Hong Kong SAR, China.

## Abstract

The health authorities reported that 3,019 Chinese health workers were infected with the severe acute respiratory syndrome coronavirus 2 (SARS-CoV-2), of whom, ten died. This article explored relevant reasons and offered suggestions to reduce the risk of infection and provide emergency psychological response for this population.

## Introduction

Recently two alarming reports have been released from the health authorities of China. On the 14 February 2020, the National Health Commission of China 1 reported that as of midnight February 11, 2020, a total of 1,716 health workers had been infected with the 2019 novel coronavirus disease (COVID-19; formerly known as 2019-nCoV) nationwide; of whom, 1,502 (87.5%) were in Hubei province, with 1,102 in Wuhan. On February 17, 2020 the China CDC Weekly 2 reported that a total of 3,019 Chinese health workers have been infected with the severe acute respiratory syndrome coronavirus 2 (SARS-CoV-2); of whom, 1,716 were confirmed with the COVID-19. As of February 20, ten health workers have died 3. The infected health workers included Dr. Chaolin Huang and his colleagues who first reported clinical features of patients infected with COVID-19 4, and the deaths included the whistleblower, Dr. Wenliang Li 5, one of the eight doctors who initially warned about the COVID-19 when it first emerged in Wuhan, who were later reprimanded by the local police for spreading rumors, and Dr. Zhiming Liu, the President of Wuhan Wuchang Hospital in Hubei province 6. In contrast, however, the local health authority in Wuhan reported that only 15 health workers were diagnosed with COVID-19 by January 21, 2020 7.

We speculate several factors that possibly account for the rapidly increased number of infected health workers. First, there was inadequate or even lack of awareness and precautionary measures combating the COVID-19 outbreak, particularly in January 2020. For example, in late January 2020, millions of people left Wuhan on the “last train home” for the Chinese New Year, which was believed later as one of the major contributing factors for the rapid COVID-19 transmission. Some individuals either infected the disease in Wuhan or on their return journey back to their home towns. These individuals sought help from local health services and thus infected the local health workers in the clinics. In addition, in late January 2020, there could be some patients with atypical symptoms in the incubation period of the COVID-19 whom consulted doctors in different clinics. Throughout the consultation, the virus was transmitted to health workers without full protective gear. Second, insufficient supplies of full protective gear to frontline health workers have inevitably exposed them to high infectious risk, particularly in primary health service. Third, the diagnostic criteria for confirmed cases with the COVID-19 have been loosened recently. The updated diagnostic criteria in China stated that suspected cases with pneumonia-related computerized tomography (CT) scan results would be counted as clinically diagnosed COVID-19 patients 8. Sudden change in diagnostic criteria may result in the rapid increase on the infected health workers figures.

In recent years, Chinese health workers are often confronted with frustrating situations in the health care system including serious workplace violence against clinicians. A meta-analysis found that the overall prevalence of workplace violence was 62.4% among Chinese health workers 9. Regardless of the unsafe clinical environment, Chinese health workers are always committed to provide timely health services without any hesitation or reservations. For instance, as of February 12, 2020, a total of 189 external expert teams comprising 21,569 health workers from other regions of China have volunteered to work in Hubei province, disregarding the high risk of contracting the infection and the high mortality rate of the COVID-19 among health workers 10.

Given the surge of increasing number of confirmed cases on a daily basis in China, health workers have to take care of the increasing number of patients flocking in hospitals. Patient overload may lead to staff burnout, physical and mental exhaustion. Some health workers infected with the COVID-19 may feel helpless, hopeless and being isolated too. Thus, health care workers' mental health may be at stake and should not be overlooked. Health authority, stakeholders and health policymakers should make concerted effort to ensure sufficient supplies of protective gear to front line health workers who are arguably at highest risk of contracting the COVID-19. More importantly, health workers should be provided with professional counselling if they showed early signs of burnout, anxiety and/ depression to minimize the risk of developing psychiatric morbidity.

It is beyond doubt that Chinese health workers deserve utmost respect, tribute and applause in the globe. Chinese health workers are simply a group of silent, loyal, committed and respectable sub- population who are always there protecting the physically ill and vulnerable people in the community at large.
